# First person – Carmen Hernández-Ainsa

**DOI:** 10.1242/dmm.049434

**Published:** 2022-03-01

## Abstract

First Person is a series of interviews with the first authors of a selection of papers published in Disease Models & Mechanisms, helping early-career researchers promote themselves alongside their papers. Carmen Hernández-Ainsa is first author on ‘ Development and characterization of cell models harbouring mtDNA deletions for *in vitr*o study of Pearson syndrome’, published in DMM. Carmen is a research assistant in the lab of Eduardo Ruiz-Pesini at Universidad de Zaragoza, Zaragoza, Spain, investigating mitochondrial DNA deletions, pathological consequences and treatments through development of new *in vitro* models.

**Figure:**
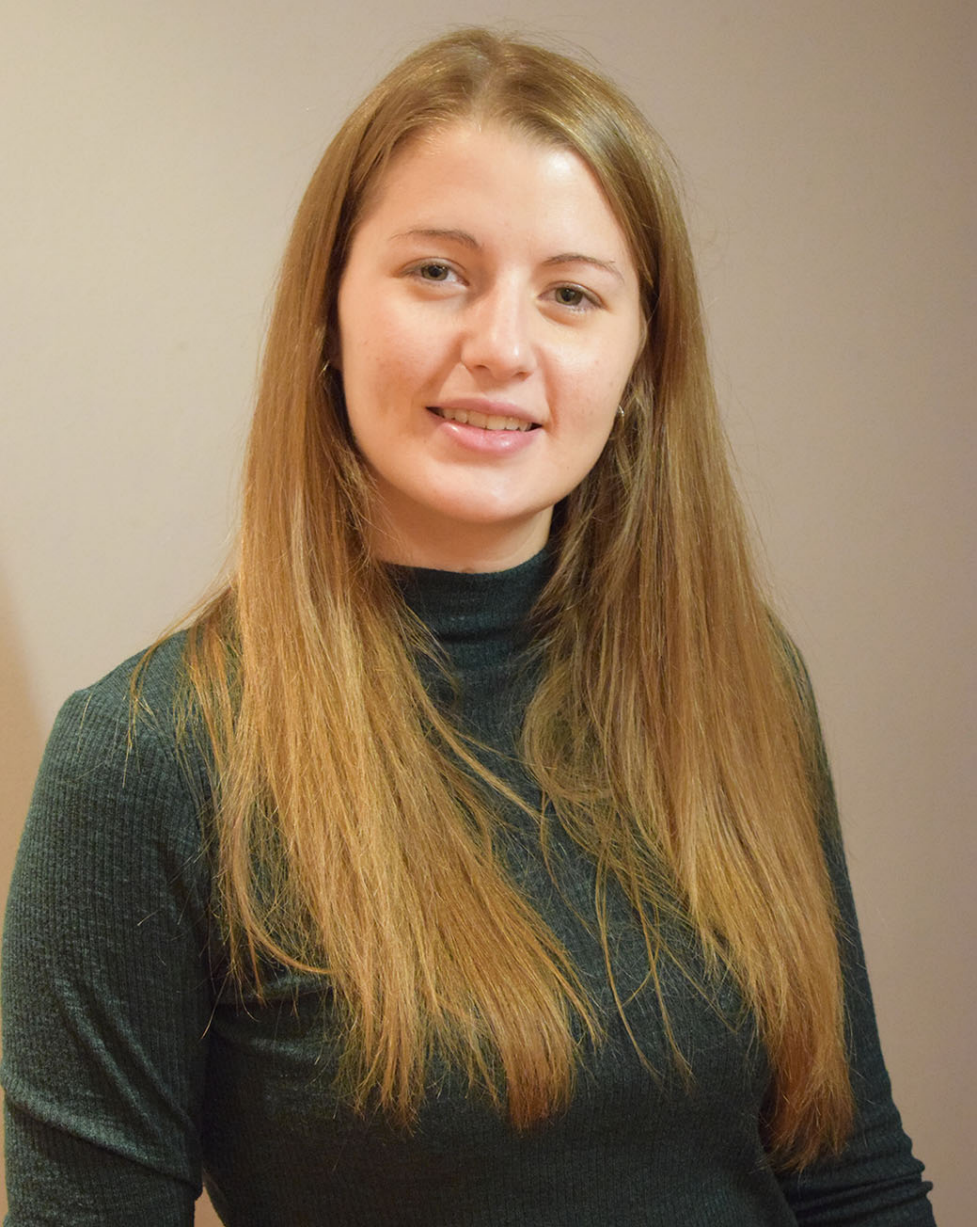
Carmen Hernández-Ainsa

**How would you explain the main findings of your paper to non-scientific family and friends?**

Pearson syndrome (PS) is a metabolic pathology mainly characterized by sideroblastic anaemia and exocrine pancreas dysfunction. It is caused by a deletion in mitochondrial DNA (mtDNA), which is located in mitochondria, considered the powerhouse of our cells. mtDNA deletion causes the loss of several mitochondrial genes, and it is normally fatal in infancy. The size, the number of deleted molecules and the specific cell type can influence the pathological consequences. PS is considered a rare disease, so related research projects are scarce, and every new piece of information is a milestone.

In this paper, we presented the development and characterization of three cell models harbouring mtDNA deletions with different metabolic profiles. We observed a significant dysfunction in all of them, but with some interesting differences that could allow a better understanding of the course of the disease. Furthermore, we have been able to establish the range of deleted mtDNA amounts that are necessary to lead to a pathological phenotype in a specific cell model. Therefore, we expect that these findings will be essential for the study of treatments in the future.

“PS is considered a rare disease, so related research projects are scarce, and every new piece of information is a milestone.”

**What are the potential implications of these results for your field of research?**

The generation of different cell models carrying the same mtDNA deletion allows us to compare its behaviour, depending on the metabolic profile. Therefore, this will have a direct impact on new treatment development. We consider that induced pluripotent stem cells (iPSCs) represent the most promising model, since they allow differentiation or generation of every cell type *in vitro*. This offers new possibilities to study the pathophysiology of PS in specific cell types frequently affected in patients, such as blood cells or pancreatic cells, as well as to approach a more specialized treatment. This point is especially important in the field of rare disease because of the limited number of patients to be studied.

**Figure:**
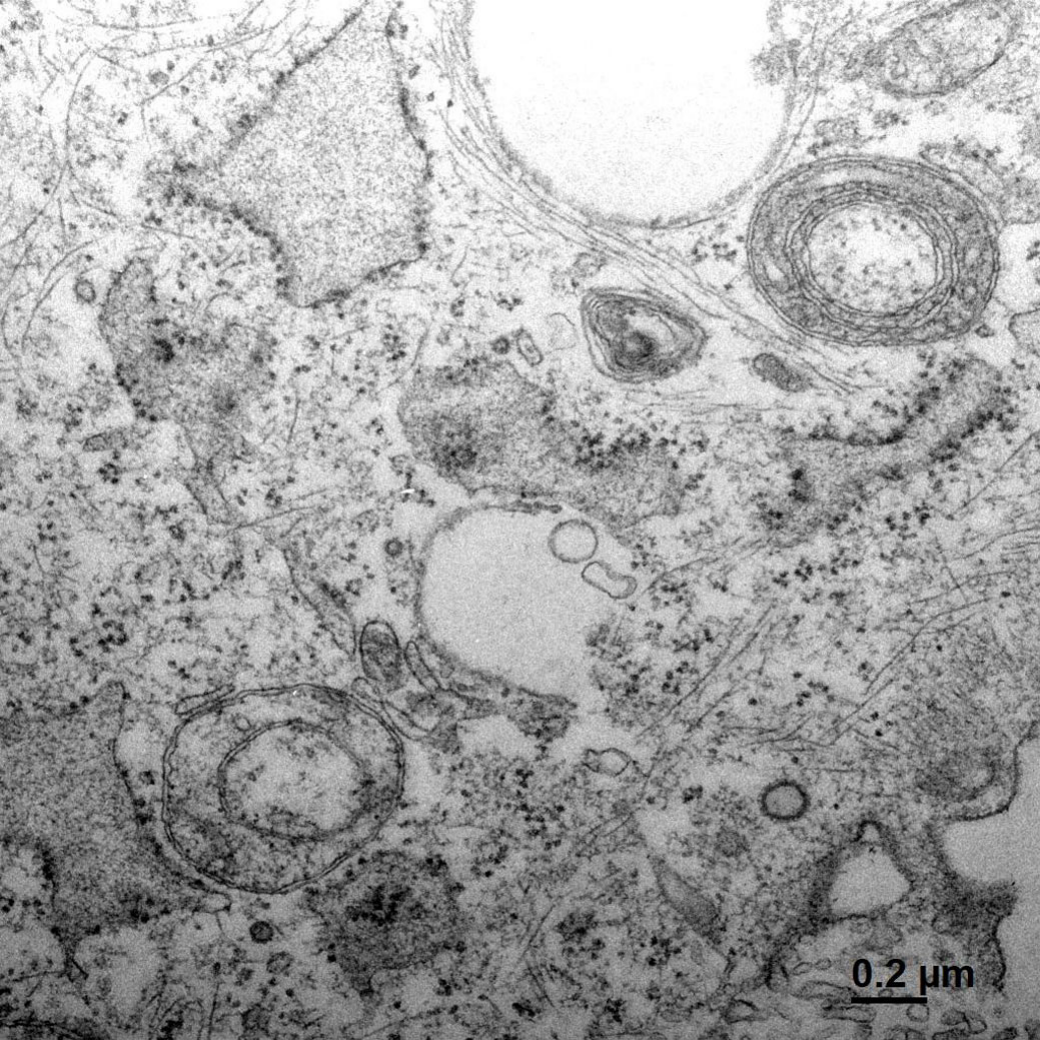
Aberrant morphology of mitochondria in fibroblasts harbouring a mtDNA deletion.

**What are the main advantages and drawbacks of the model system you have used as it relates to the disease you are investigating?**

As mentioned, the incidence of this disease is very low, so the possibility of studying its pathological mechanisms in every specific cell type generated from iPSCs is an enormous advantage. Besides, two of three cell models presented in this work (fibroblasts and iPSCs), and potential differentiated cells generated from the latter, are euploid and conserve the nuclear and mitochondrial genetic background of the original patient, thus allowing a better approach to the real response to a treatment. On the other hand, despite cybrids presenting an aneuploid karyotype, they allow us to clearly observe the effect of mtDNA deletion size and amount on the phenotype regardless of nuclear background. This has been the reason for the importance of this model in the mitochondrial research field since many years ago.

The main drawback of these models is their manipulation and generation, which can be complicated, time consuming and expensive, especially the iPSC model, which could delay the development of a personalized treatment.

**What has surprised you the most while conducting your research?**

After many difficulties to obtain iPSCs carrying a mtDNA deletion, we were surprised that they were able to proliferate and differentiate correctly despite harbouring a high heteroplasmy level. Moreover, during biochemical analysis of the different cell models, we expected to observe a mitochondrial biogenesis triggered by the mtDNA deletion in all of them. However, we obtained interesting differences, probably due to their different metabolic profile, which will be attractive for further studies.

**Describe what you think is the most significant challenge impacting your research at this time and how will this be addressed over the next 10 years?**

The final objective of our research is achieving an effective treatment for PS patients, but we know that this is still a challenge due to the lack of knowledge of this disease. This work aims to be the beginning of a research line that should be continued. We have developed different tools that open a new window in this field. The next step is to achieve optimized protocols to generate all types of human cells *in vitro* from iPSCs in order to study specific pathophysiologic mechanisms and to develop correct therapies to solve them.

**What changes do you think could improve the professional lives of early-career scientists?**

Governments should worry more about the current state of research and scientists. The pandemic has highlighted the importance of science for society, and it has demonstrated that scientists can achieve impressive goals with enough money and resources. However, the budget allocated by governments to science is scarce, which slows down the development of many research projects.

**What's next for you?**

I would like to continue to research PS, working with the cell models we have developed, trying to get some of the affected cell types *in vitro* to increase knowledge of this disease and test new treatments.
